# Absorption and Tissue Distribution of Environmental Pollutant HFPO-DA, and Its Effect on Hepatic Lipid Metabolism Reprogramming in Mice

**DOI:** 10.3390/toxics13100850

**Published:** 2025-10-08

**Authors:** Jie Peng, Wei Jiang, Zi Long, Yueying Cui, Guizhen Zhu, Rui Liu, Deqin Kong, Weihua Yu, Yuliang Li, Chunxu Hai

**Affiliations:** 1 Department of Toxicology, The Shaanxi Provincial Key Laboratory of Free Radical Biology and Medicine, The Shaanxi Provincial Key Laboratory of Environmental Health Hazard Assessment and Protection, The Ministry of Education Key Lab of Hazard Assessment and Control in Special Operational Environment, School of Public Health, The Fourth Military Medical University, Xi’an 710032, China; 19991205301@163.com (J.P.); liurui123@fmmu.edu.cn (R.L.); kongdeqin@fmmu.edu.cn (D.K.); yuweihua1@fmmu.edu.cn (W.Y.); 2 Department of Clinical Medicine Laboratory, Air Force Medical Center, The Fourth Military Medical University, Beijing 100142, China; cyygsjyg@163.com (Y.C.); 13121437811@163.com (G.Z.); 3 Department of Pharmacy, The Second Affiliated Hospital, The Fourth Military Medical University, Xi’an 710038, China; aact@163.com; 4 Department of Aeromedical Support & Flight Safety, Air Force Medical Center, The Fourth Military Medical University, Beijing 100142, China; luoze0317@126.com

**Keywords:** HFPO-DA, pharmacokinetics, tissue distribution, lipid metabolism, UPLC-HRMS

## Abstract

Objective: Hexafluoropropylene oxide dimer acid (HFPO-DA), also known as GenX, is widely used globally, raising concerns about its safety and public health implications. However, its toxicity mechanism remains unclear. The purpose of this study was to develop a reliable method for detecting HFPO-DA in mice and to investigate its absorption, distribution, and impact on hepatic lipid metabolism. Method: HFPO-DA levels were measured in the serum and eight tissues of C57BL/6J mice after oral administration using ultra-performance liquid chromatography–tandem mass spectrometry (UPLC-MS/MS). Lipid metabolites in the liver were also detected and analyzed. Results: HFPO-DA was rapidly absorbed into the bloodstream and widely distributed throughout all tested tissues. It penetrated the blood–brain barrier, with the highest concentration in the liver; however, long-term effects on the lungs also warrant attention. HFPO-DA disrupted liver lipid metabolism, leading to acylcarnitine accumulation while lowering triglycerides and cholesterol. Conclusion: This study on the pharmacokinetics and tissue distribution of HFPO-DA in mice following oral exposure revealed that HFPO-DA exacerbates liver injury by altering hepatic lipid metabolism. These findings provide theoretical support for toxicological studies on the emerging environmental pollutant HFPO-DA.

## 1. Introduction

Perfluoroalkyl and polyfluoroalkyl substances (PFAS), with classic representatives perfluorooctanoic acid (PFOA) and perfluorooctane sulfonic acid (PFOS), are widely used in coatings, cosmetics, clothing, electronic devices, etc., due to their unique properties. However, due to their bioaccumulation and multi-organ toxicity, these substances have become significant threats to both the environment and human health and are restricted or banned under the Stockholm Convention [1]. Recently, hexafluoropropylene oxide dimer acid (HFPO-DA), also known as GenX, which has similar physicochemical properties but a more rapid rate of environmental degradation, has developed as an alternative to PFAS in production and use [2]. As the production and use of HFPO-DA have increased rapidly, water pollution has become the most concerning issue. HFPO-DA has been detected in many rivers in various countries, with concentrations reaching up to 9350 ng·L^−1^ and possibly rising [3,4]. HFPO-DA has strong migration capabilities, and as water pollution expands, HFPO-DA has been detected in soil, aquatic organisms, plants, and animals [5]. In two epidemiological cohort studies conducted in Anniston, USA, in 2005 and 2014, HFPO-DA was detected in human serum, with the detection rate doubling from 4.4% to 8.9% over time [6]. A study in China found that HFPO-DA is significantly linked to an increased risk of unexplained recurrent miscarriages [7]. Therefore, HFPO-DA has already been shown to affect human health and is emerging as a new public health concern.

Exploring the absorption and distribution of toxic substances within the body is crucial for understanding their toxic effects. To date, data on the absorption and distribution of HFPO-DA in mice are limited. Studies have demonstrated that HFPO-DA exhibits greater toxicity than PFOA at the same internal dose [8,9]. It can cause more severe oxidative damage, affecting various tissues and organs; for example, neurotoxicity, cardiovascular and reproductive toxicity in zebrafish, hepatotoxicity in rats, and effects on gene expression in thyroid cells [10,11,12,13]. Chronic toxicity studies indicate that HFPO-DA causes varying degrees of damage to different tissues [14], suggesting that HFPO-DA may have differential tissue accumulation or affinity. HFPO-DA is eliminated from the body more quickly than PFOA. It is rapidly excreted in rats, mice, and monkeys without metabolism, leaving the body in its original form via urine [15]. The distribution of HFPO-DA among various tissues before excretion, as well as its absorption and excretion by these tissues, is not yet fully understood. Previous methods for determining HFPO-DA content have primarily focused on analyzing environmental samples, including rivers, drinking water, soil, and surface sediments. However, measurements of biological samples are relatively rare [4,16].

The mechanism by which HFPO-DA causes liver damage remains unclear. Transcriptome analysis revealed that short-chain PFAS are significantly enriched in fatty acid metabolism and β-oxidation pathways, with the expression of related genes being upregulated [17]. The detection of HFPO-DA revealed a significant enrichment of the PPARα signaling pathway in both the gene expression profiles of male neonatal mouse livers and the transcriptome analysis of liver cells exposed in vitro [18,19,20]. The observed hepatotoxic effects to date are also broadly consistent with the activation of the PPARα pathway, such as increased liver weight, hepatomegaly, and increased cell proliferation [21,22,23]. However, some studies have shown that HFPO-DA increases triglyceride content in mouse liver and elevates triglycerides and cholesterol in neonatal mouse serum [24]. Other studies have found inconsistent triglyceride changes in serum and liver [25]. The PPARα signaling pathway is closely linked to lipid disorders in various diseases and may coordinate and promote the expression of many target genes involved in fatty acid metabolism [26,27,28,29]. Therefore, it is necessary to explore the mode of action of HFPO-DA from the perspective of lipid metabolism by analyzing its underlying mechanisms and relationship with the PPARα pathway.

This study aimed to elucidate the absorption and tissue distribution of HFPO-DA in mice, identify target organs for toxic effects, and assess the impact of HFPO-DA on lipid metabolism in target organs. An ultra-performance liquid chromatography–tandem mass spectrometry (UPLC-HRMS) method for the analysis of HFPO-DA in biological samples was developed. The HFPO-DA content in various mouse tissues was measured following a single oral exposure for 24 h. Additionally, liver lipid content in mice was assessed after 16 days of continuous oral HFPO-DA administration. Potential toxic effects were evaluated through tissue action concentrations and lipid metabolome profiling. This study highlights the detrimental effects of HFPO-DA on mice and offers a theoretical foundation for assessing risks associated with HFPO-DA.

## 2. Materials and Methods

### 2.1. Chemicals

The HFPO-DA standard (Lot No.M29A11K123022; purity: 99.9%) was procured from Shanghai Yuanye Bio-Technology Co., Ltd. (Shanghai, China). Chromatography-grade methanol and acetonitrile were purchased from Aladdin Reagent (Shanghai) Co., Ltd. (Shanghai, China). The sodium chloride (0.9%) injectable was purchased from Wuhan Servicebio Technology Co., Ltd. (Wuhan, China). The experimental process utilized ultrapure water from a Milli-Q Reagent Water System (Merck Millipore, Burlington, MA, USA).

### 2.2. Stock Solutions, Reference Standards, and Quality Control (QC) Samples

The HFPO-DA standard was dissolved in methanol to prepare a 16 mg·mL^−1^ stock solution. The solution was then diluted to concentrations of 1.6, 4, 8, 16, 40, 80, 160, 400, 800, 1600, 4000, and 8000 ng·mL^−1^ for use. A working solution of 12 µg·mL^−1^ hydroxymethyl coumarin (internal standard, IS) was prepared according to the same procedure. To validate the method’s effectiveness, control samples were prepared using the same blank matrix as the test samples, with HFPO-DA concentrations of 6, 400, and 800 ng·mL^−1^, designated as low, medium, and high levels, respectively.

### 2.3. Animals and Chemical Treatment

Clean-grade C57BL/6J mice, 8 weeks old, half female and half male, were supplied by SPF (Beijing) Biotechnology Co., Ltd. (Beijing, China) The animals were housed in the laboratory animal center, where they were subjected to a 12 h light/dark cycle. They had free access to water and standard food. Prior to the commencement of the experiment, the animals were permitted a period of acclimation spanning one week. During the experiment, the subjects were placed in a quiet room with a temperature range of 15 to 25 °C and a humidity range of 55% to 65%. The protocol for the experimental animals was approved by the Ethics Committee of Tangdu Hospital, Fourth Military Medical University (approval number GKJ-Y-202503-128, dated 5 March 2025).

A total of 72 mice were used for the single-dose oral exposure experiment and the multiple-dose oral exposure experiment. In accordance with ICH S3A and S3R guidelines, groups were formed using GraphPad Prism’s randomization tool, with six animals per group, comprising three males and three females. The cages were numbered by technicians not involved in the experiment, and the experimenters remained unaware of the group assignments. Before the experiment commenced, the mice were subjected to a 12 h fast, during which they had access to water but not to food. During the experimental period, the mice’s condition was closely monitored. If weight loss exceeded 20% or severe respiratory distress occurred, the mice were euthanized using CO_2_ inhalation, and the incident was reported.

Single-dose oral exposure experiment: Sixty mice were randomly divided into ten groups. In the experiment, mice received an oral dose of 30 mg·kg^−1^ HFPO-DA dissolved in water. Multiple-dose oral exposure experiment: Twelve mice were selected and randomly divided into a control group and an experimental group (HFPO-DA). The experimental group received 3 mg·kg^−1^ of HFPO-DA aqueous solution orally, while the control group received an equivalent quantity of distilled water. Oral administration continued for 16 days, once daily.

Mice were anesthetized with isoflurane at 0, 0.0833, 0.25, 0.5, 1, 2, 4, 8, 12, and 24 h (single-dose oral exposure) and 17 days (multiple-dose oral exposure) to collect samples for analysis. Blood was extracted from the inferior vena cava, centrifuged at 3000 rpm for 10 min, and the serum present within the supernatant was collected for subsequent processing. Additionally, tissue samples were collected from various anatomical sites—including the heart, brain, lung, spleen, liver, kidney, epididymal/gonadal white adipose tissue (WAT), and interscapular brown adipose tissue (BAT)—for determining the HFPO-DA content, as well as the content of lipid metabolites in the liver. The body weight and liver weight of mice in the multiple-dose oral exposure experiment were determined on day 17. The tissues were rinsed three times with double-distilled water, and excess water was absorbed using filter paper. All specimens were stored at −80 °C until analysis.

### 2.4. Sample Preparation

Processing samples for HFPO-DA detection: The mouse serum samples were removed from −80 °C storage, allowed to thaw at 25 °C, and then mixed at 25 °C using a vortex mixer. Tissue samples were removed from −80 °C storage and allowed to thaw at 25 °C. Place each tissue sample into a pre-chilled 2-milliliter grinding tube containing grinding beads. Add purified water at a tissue-to-water ratio of 1:6 (*w*/*v*). Using a tissue grinder (Scientz-48, Servicebio, China), intermittent grinding (grind for 30 s, pause for 30 s) was performed at 25 Hz and 4° C for three cycles. The homogenate was centrifuged at 4 °C and 12,000 rpm for 15 min, and the resulting supernatant was collected. All samples were prepared using the methanol protein precipitation method. A sample (100 µL) was mixed with internal standard solution (20 µL, containing 12 µg·mL^−1^ hydroxymethyl coumarin) and methanol (300 µL). Subsequently, the samples were mixed by vortex at 5000 rpm for 1 min, then centrifuged at 4 °C and 12,000 rpm for 15 min. The supernatant was removed and analyzed by UPLC-MS/MS.

Processing samples for lipid detection: The liver tissue samples were removed from −80 °C storage and thawed on ice. A sample (20 mg) was placed into a 2 mL centrifuge tube, steel beads were added, and the sample was homogenized for 20 s using a ball mill. The sample was then centrifuged at 4 °C and 3000 rpm for 30 s. Internal standard lipid extraction solution (1 mL, 3:1 (*v*/*v*) tert-butyl methyl ether: methanol) was added, and the resulting mixture was vortexed for 15 min. Then, water (200 μL) was added, and the sample was vortex mixed at 4 °C and then centrifuged at 12,000 rpm for 15 min. A 200 μL aliquot of the supernatant was evaporated to dryness, and lipid resuspension solution (200 μL, 1:1 (*v*/*v*) acetonitrile: isopropanol) was added. The sample was thoroughly vortex mixed for 5 min, then centrifuged at 12,000 rpm for 5 min. The resulting mixture was analyzed by UPLC-MS/MS.

### 2.5. Analytical Instruments and Conditions

HFPO-DA detection: Data collection was primarily via UPLC (LC-30, Shimadzu, Kyoto, Japan) and MS/MS (QTRAP 5500, AB Sciex, Framingham, MA, USA), equipped with an electrospray ionization (ESI) source. HFPO-DA and the internal standard were separated using an Agilent Poroshell 120 EC-C18 column (4.6 × 100 mm, 2.7 μm; Agilent Technologies, Santa Clara, CA, USA). The mobile phase comprised acetonitrile (phase A) and water with 0.1% formic acid (phase B). The flow rate was 0.4 mL·min^−1^, the column temperature was maintained at 25 °C, and the injection volume was 5 μL. The automatic sampler was maintained at 4 °C during analysis. The elution system was set for a continuous duration of 7 min with the following gradient: 30% mobile phase A from 0 to 1 min, followed by a gradient to 80% mobile phase A from 1 to 5 min, then a gradient back to 30% mobile phase A from 5 to 5.5 min, and finally 30% mobile phase A from 5.5 to 7 min.

ESI is a suitable method for positive and negative ion scanning when combined with multiple reaction monitoring (MRM) for ion detection. The conditions were as follows. HFPO-DA, *m*/*z* 175.0–132.6: declustering potential (DP), 20 V; collision energy (CE), 7 V. Hydroxymethylcoumarin (IS), *m*/*z* 328.8–284.8: DP, 73 V; CE, 27 V. The ion source temperature was 500 °C, the curtain gas pressure was maintained at 30 psi, and the collision gas level was medium. The ion voltage calibration was −4500 V (negative ion mode), and the pressure of both the sprayer gas and the auxiliary heating gas was 50 psi.

Lipid metabolite detection: Data collection was primarily via UPLC (ExionLC AD, AB Sciex, Framingham, MA, USA) and MS/MS (QTRAP 5500, AB Sciex, Framingham, MA, USA), equipped with an electrospray ionization (ESI) source. A Poroshell Thermo Accucore™ C30 column (2.1 × 100 mm, 2.6 μm) was utilized for the separation process. The mobile phase comprised 60% acetonitrile and 40% water, with 0.1% formic acid (phase A), and 10% acetonitrile and 90% isopropanol, with 0.1% formic acid (phase B). The flow rate was 0.35 mL/min^−1^, and the operating temperature was 45 °C. The elution system used the following gradient: Initially (at 0 min), the mobile phase was 80% mobile phase A, followed by a gradient to 70% mobile phase A from 0 to 2 min, then further gradients to 40% mobile phase A by 3 min, 15% mobile phase A by 9 min, 10% mobile phase A by 14 min, and 5% mobile phase A by 15.5 min. The 5% mobile phase A composition was maintained until 17.3 min, then was restored to the initial 80% mobile phase A at 17.5 min.

Linear ion trap (LIT) and triple quadrupole (QQQ) scans were acquired on a QQQ-LIT mass spectrometer (QTRAP). The ESI source operation parameters were as follows: source temperature, 500 °C; ion spray voltage (IS), 5500 V (positive), −4500 V (negative); ion source gas 1 (GS1), gas 2 (GS2), curtain gas (CUR) at 45, 55, and 35 psi, respectively; collision gas (CAD), medium. Instrument tuning and mass calibration were performed with 10 and 100 μM polypropylene glycol solutions in QQQ and LIT modes, respectively. QQQ scans were acquired as MRM experiments with the collision gas (nitrogen) set to 5 psi. DP and CE for individual MRM transitions were performed, followed by further optimization of DP and CE.

### 2.6. Method Validation

#### 2.6.1. Selectivity

The selectivity of the method was evaluated using blank mouse serum samples, samples with added HFPO-DA standard and IS, and serum samples collected 5 min after oral administration of HFPO-DA (30 mg·kg^−1^). This was to ensure that endogenous components did not interfere with the analyte and IS. Tissue sample processing was similar to serum sample processing.

#### 2.6.2. Linearity and Lower Limit of Quantitation (LLOQ)

Different concentrations of HFPO-DA were added to blank mouse serum or tissue homogenates containing internal standards. These samples were processed according to the sample preparation method, yielding the following final calibration concentrations of HFPO-DA: 0.4, 1, 2, 4, 10, 20, 40, 100, 200, 400, 1000, and 2000 ng·mL^−1^. These samples were then analyzed. A standard curve of HFPO-DA concentration (X) versus peak area (Y) was established. The signal-to-noise ratio (S/N) for the lower limit of quantitation (LLOQ) exceeded 10. Serum and tissue homogenate samples with concentrations exceeding the linear range were diluted with physiological saline or distilled water until the measured values fell within the linear range, then sample processing and detection were continued.

#### 2.6.3. Precision and Accuracy

To assess the accuracy and precision of the measurements, intra-day and inter-day variations in the samples were examined. QC samples of HFPO-DA were configured at three concentrations (low, medium, and high level), with six replicates for each concentration. All samples were measured once daily for three consecutive days. Intra-day variation was calculated by comparing samples within a single day, while inter-day variation was calculated by comparing samples over the three days. Precision was ascertained by estimating the relative standard deviation (RSD), while accuracy was denoted by the relative error (RE). Tissue sample processing was similar to serum sample processing.

#### 2.6.4. Matrix Effect and Extraction Recovery Rate

Samples of HFPO-DA at high, medium, and low concentrations were prepared in blank mouse serum, pure water, and methanol. The peak areas of HFPO-DA were recorded in the following samples. (a) Standard serum matrix sample: Standard was added to the blank serum matrix, and then the sample processing procedure was followed to precipitate proteins and collect the supernatant. (b) Matrix-treated samples: In the blank serum matrix, proteins were precipitated by following the sample processing procedure. The supernatant was collected, and then the standard sample was added. (c) Methanol samples: Standard solutions of various concentrations were prepared directly in methanol. The extraction recoveries were determined by calculating the ratio of (a) to (c). The matrix effect was determined by calculating the ratio of (b) to (c). Tissue sample processing was similar to serum sample processing.

#### 2.6.5. Stability

Samples with high, medium, and low concentrations of HFPO-DA were prepared from blank mouse serum. Subsequently, the HFPO-DA content of the samples was evaluated after three different treatments: initial storage at ambient temperature for 5 h, storage at −80 °C for 15 days, and three freeze–thaw cycles (−80 °C for 3 h followed by 25 °C for 3 h per cycle). The mean concentration and standard deviation were calculated for the samples, as well as the RE value of the deviation between the measured and nominal concentrations. Tissue sample processing was similar to serum sample processing.

#### 2.6.6. Dilution Integrity

Mouse serum samples were prepared with high concentrations of HFPO-DA, using the appropriate dilution of blank serum to adjust the concentration. The absolute value of the RE between the measured diluted concentration and the product of the dilution factor and the expected theoretical value was required to be less than 15% to ensure the reliability and accuracy of the concentration data obtained through dilution. Tissue sample processing was similar to serum sample processing.

### 2.7. Statistical Analysis

The subsequent analysis utilized the Process MS module in Analyst 1.6.3. Phoenix WinNonlin software (version 8.3.5) was used to fit blood concentration data and plot concentration-time curves, applying non-compartmental model analysis (NCA) for pharmacokinetic parameter analysis. The statistical analysis of the lipid metabolite data was conducted using R software (version 4.3.2). The data were graphed using GraphPad Prism 9, and *t*-tests were performed using SPSS 27.0. A statistically significant result was where *p* < 0.05.

## 3. Results

### 3.1. Method Validation

#### 3.1.1. Selectivity

**Figure 1:**
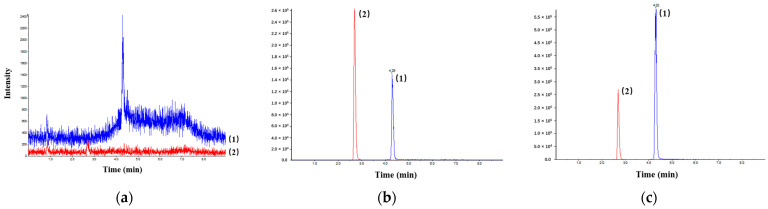
UPLC-HRMS of HFPO-DA (1) and IS (2) in (**a**) blank mouse serum; (**b**) mouse serum containing HFPO-DA and IS; (**c**) samples collected from mice 5 min after oral administration of HFPO-DA aqueous solution.

The serum and tissue sample HFPO-DA measurements were performed using UPLC-MS/MS. The HFPO-DA and IS chromatograms of serum are displayed in Figure 1. These confirmed reasonable specificity for HFPO-DA, and that impurities did not interfere with the accurate measurement of its concentration.

#### 3.1.2. Linearity and LLOQ

The linear regression equation and LLOQ of HFPO-DA in serum are shown in Table 1. These results demonstrated that HFPO-DA exhibited adequate linearity within the concentration range of 2 to 1000 ng·mL^−1^. Additionally, the quantification, accuracy, and precision limits were all within the requisite range, thereby substantiating the method’s applicability to pharmacokinetic and tissue distribution studies.

#### 3.1.3. Precision and Accuracy

Table 2 shows the precision and accuracy results for serum HFPO-DA detection. The intra-day and inter-day precision RSD values were both less than 15%. The intra-day accuracy RE was −1.43% to 14.10% and the inter-day accuracy RE was −2.27% to 0.01%. These findings suggested that this method exhibited adequate precision and accuracy in measuring HFPO-DA concentrations.

#### 3.1.4. Matrix Effect and Extraction Recovery Rate

The extraction recovery rates and matrix effects at varying HFPO-DA concentration levels (low, medium, and high) are presented in Table 3. The HFPO-DA extraction recovery rate in mouse serum ranged from 1.35% to 3.31%, indicating stability and repeatability. The matrix effect ranged from 1.29% to 2.24%, indicating that the influence of endogenous sample components was minimal.

#### 3.1.5. Stability

The stability correlation analysis of HFPO-DA under different storage conditions is presented in Table 4. The HFPO-DA precision RSD was less than 15%, and the RE accuracy ranged from −2.62% to 14.71%. These results indicated that HFPO-DA was stable under various storage conditions.

#### 3.1.6. Dilution Integrity

The impact of dilution on the measurements is shown in Table 5. The results indicate that diluting high-concentration samples 5-fold, 10-fold, and 25-fold with blank mouse serum did not affect the measurement of HFPO-DA.

The methodological validation results for heart, liver, spleen, lung, kidney, brain, and adipose tissue are presented in Tables S1–S5. The method exhibited high specificity, excellent extraction recovery, and stability, with matrix effects effectively managed. Thus, it met the requirements for the quantitative analysis of biological samples.

### 3.2. Pharmacokinetic Study in Mice

**Figure 2:**
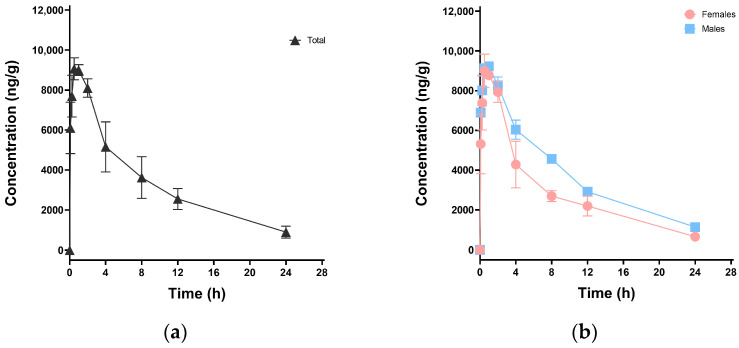
Mean HFPO-DA concentration-time curves in mouse serum after oral exposure to HFPO-DA at 30 mg·kg^−1^: (**a**) all mice; (**b**) females and males.

After oral administration of 30 mg·kg^−1^ HFPO-DA to mice, serum was collected for extraction and analysis. A concentration-time curve was plotted for HFPO-DA in mouse serum, as shown in Figure 2. The pharmacokinetic parameters were derived from sparse data NCA using Phoenix WinNonlin software (version 8.3.5), as outlined in Table 6. HFPO-DA was quickly absorbed in mice, reaching a peak concentration (C_max_) of 9066.67 ng·mL^−1^ in an average of 0.5 h (T_max_). The elimination half-life (t_1/2_) was 7.95 h, and the clearance rate (CL_z/F_) was 330.06 mL·h^−1^·kg^−1^. HFPO-DA showed extensive tissue distribution, with an apparent volume of distribution (V_d_) of 3783.25 mL·kg^−1^, indicating possible tissue accumulation. Initially, differences in the dynamic metabolism of HFPO-DA were observed between male and female mice. The results showed that males generally had higher C_max_ and T_1/2_ values, as well as a larger area under the curve (AUC_0-t_). For a more detailed exploration of this phenomenon, a larger sample size is needed.

### 3.3. Tissue Distribution in Mice

#### 3.3.1. Single-Dose Oral Exposure Tissue Distribution

**Figure 3:**
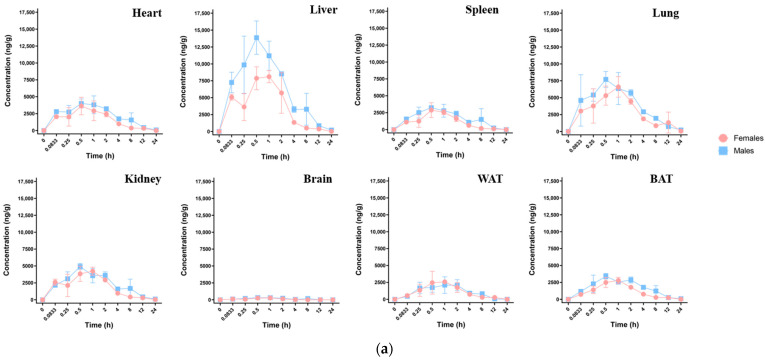

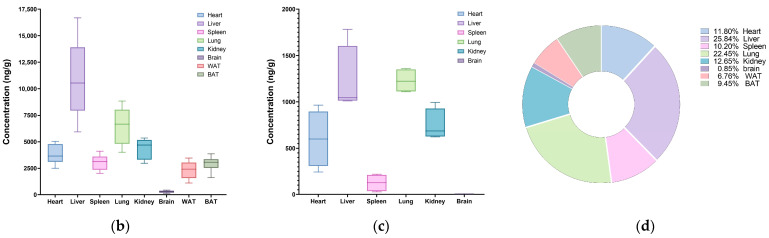
Tissue HFPO-DA distribution in mice: (**a**) Mean concentration-time curve in mouse tissue after oral exposure at 30 mg·kg^−1^; (**b**) concentration of HFPO-DA in mouse tissues at peak time (T_max_) after oral exposure at 30 mg·kg^−1^; (**c**) concentration of HFPO-DA in mouse tissues after 16 d of continuous exposure at 3 mg·kg^−1^; (**d**) AUC comparison of HFPO-DA in mouse tissues following oral administration at 30 mg·kg^−1^.

After oral exposure in mice, HFPO-DA was detected in eight different tissues. As illustrated in Figure 3a, the concentration-time distribution curves varied across different tissues. The absorption and distribution processes within each tissue type are shown in Table 7. HFPO-DA was detected in all tissues 0.0833 h after exposure, with T_max_ ranging from 0.5 to 1 h across different tissues, indicating the rapid and widespread distribution of HFPO-DA within the mouse body. The C_max_ (ng·g^−1^) for each tissue is shown in Figure 3b and was as follows: liver > lung > kidney > heart > spleen > brown fat > white fat > brain. The concentration in liver tissue was significantly higher than in other tissues. The concentrations in brown fat and white fat tissue were slightly lower than those in the heart and spleen, indicating that HFPO-DA has good lipophilicity. Despite its low concentration in the brain—approximately 1/40 of that found in the liver—its presence demonstrated that HFPO-DA could traverse the blood–brain barrier. The T_1/2_ in most tissues ranged from 3.5 to 4.8 h, with faster clearance observed in the spleen (2.7 h) and white fat (1.9 h). The tissue AUC_0-t_ (h·ng·g^−1^) for HFPO-DA was as follows: liver > lung > kidney > heart > spleen > brown fat > white fat > brain, as shown in Figure 3d. The mean residence time (MRT_0-t_, h·ng·g^−1^) was as follows: lung > heart > kidney > brown fat > spleen > liver > white fat > brain. Males and females exhibited differences in tissue HFPO-DA concentrations. Males had lower T_max_ values than females in the liver, lungs, kidneys, brain, and brown fat, with HFPO-DA concentrations peaking faster in males. Additionally, males generally had higher C_max_, T_1/2_, and AUC_0-t_ values, as shown in Table 7, suggesting that HFPO-DA may have a more pronounced toxic effect on male mice.

#### 3.3.2. Multiple-Dose Oral Exposure Tissue Distribution

**Figure 4:**
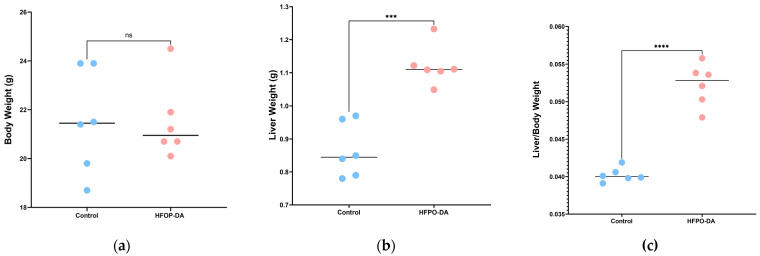
Effect of HFPO-DA on mouse liver after 16 d of continuous exposure at 3 mg·kg^−1^. The control group was administered pure water. (**a**) Body weight; (**b**) liver weight; (**c**) liver-to-body weight ratio. *** *p* < 0.001, **** *p* < 0.0001 compared with the control.

The liver-to-body weight ratio, or the ratio of liver weight to total body weight, was calculated by weighing the mice and their livers and assessing changes in liver weight. The results are shown in Figure 4. After 16 days of continuous oral exposure to HFPO-DA, the body weight of the mice did not change significantly, but their liver weight increased significantly. The liver-to-body weight ratio of rodents subjected to HFPO-DA was considerably higher than that of the control group, suggesting hepatic impairment. The concentration of HFPO-DA in various tissues is shown in Figure 3c. The HFPO-DA concentration was highest in the lung, followed by the liver, kidney, heart, and spleen. Lung tissue had the highest median concentration, closely followed by liver tissue. The distribution in kidney, heart, and spleen tissue was similar to that after a single dose of HFPO-DA. No HFPO-DA was detected in the brain tissue.

#### 3.3.3. Characteristics of Liver Lipidome Following Multiple-Dose Oral Exposure

**Figure 5:**
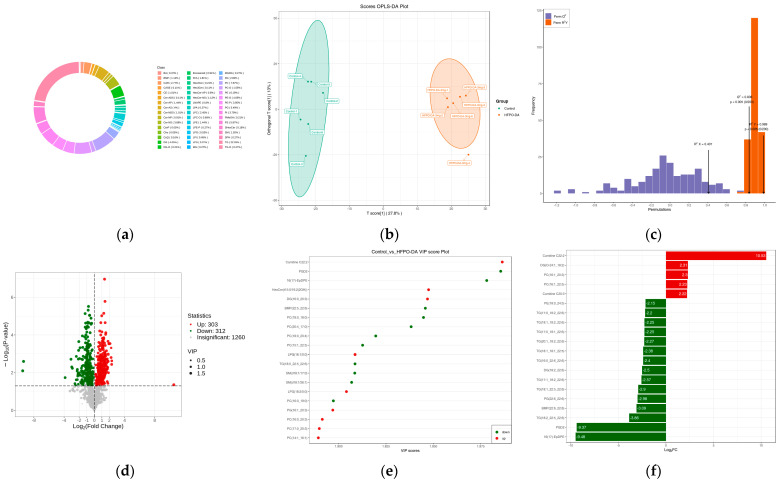
Liver lipidome after 16 d of continuous exposure to 3 mg·kg^−1^ HFPO-DA: (**a**) circular diagram of lipid subclass composition; (**b**) OPLS-DA analysis of control vs. HFPO-DA; (**c**) OPLS-DA verification in control vs. HFPO-DA; (**d**) volcano plot of differentially expressed lipids in control vs. HFPO-DA; (**e**) analysis of differentially expressed lipid VIP values in control vs. HFPO-DA; (**f**) difference multiple analysis in control vs. HFPO-DA. Red indicates lipids that increased significantly; green indicates lipids that decreased significantly; gray dots represent lipids that did not change significantly.

A targeted lipidomics analysis was performed to examine the changes in lipid metabolism in mouse liver samples after oral exposure to HFPO-DA. A total of 1875 lipid metabolites across seven major categories and 43 subcategories were detected in the mouse liver. Triglycerides (TG) comprised the most abundant category, with 413 molecules. Phosphatidylcholines (PC) and phosphatidylethanolamines (PE) were the second most abundant category, with 142 and 116 molecules, respectively. To comprehensively evaluate the lipid profile after HFPO-DA exposure, an OPLS-DA model was developed using all identified lipids (R2X = 0.407, R2Y(cum) = 0.989, and Q2(cum) = 0.836). This analysis revealed significant differences in lipid metabolism between the control group and the group exposed to HFPO-DA. Differentially expressed lipids were screened according to VIP > 1 and *p* < 0.05, identifying 615 differentially expressed lipid metabolites between the control and HFPO-DA groups. In the HFPO-DA-exposed group, 312 lipid metabolites were downregulated, while 303 were upregulated. The top five upregulated lipids were carnitine C22:2, dihydroxyacetone phosphate (DG, O-24:1_18:2), phosphatidylserine (PS, 15:0_20:3), PC (16:1_22:5), and carnitine C20:3, two of which are long-chain acylcarnitines (LCACs). The top five downregulated lipids were 16(17)-epoxy docosapentaenoic acid (16(17)-EpDPE), prostaglandin D2 (PGD2), TG (18:2_22:5_22:6), bismonoacylglycerol phosphate (BMP, 22:6_22:6), and phosphatidylglycerol (PG, 22:6_22:6), two of which are triglycerides. The top 20 lipids, as determined by VIP values, are shown in Figure 5e. The differentially expressed top five lipid metabolites were carnitine C22:2, PGD2, 16(17)-EpDPE, hexosylceramides (HexCer, t15:0/16:2(2OH)), and DG (16:0_20:3). Further exploration revealed that exposure to HFPO-DA significantly increased acylcarnitines, mainly medium to LCAC (C10-C22), and L-carnitine also significantly increased. Most of the significantly increased fatty acids were long-chain fatty acids (LCFAs, C16-C20). A decrease in triglycerides and cholesterol esters also occurred following exposure to HFPO-DA, along with a noticeable decline in free cholesterol.

**Figure 6:**
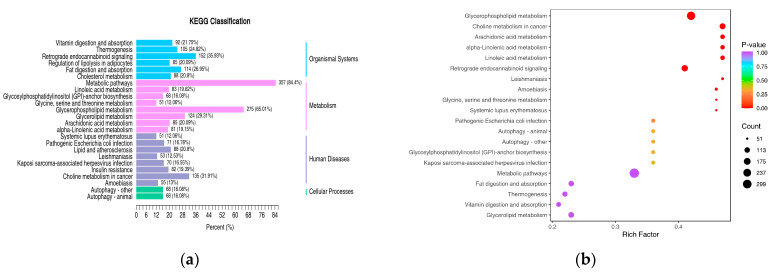
KEGG enrichment map of lipid metabolism pathways for significantly differentially expressed lipids. (**a**): Differentially expressed lipid KEGG classification analysis in control vs. HFPO-DA. (**b**): Differentially expressed lipid KEGG enrichment analysis in control vs. HFPO-DA.

KEGG enrichment analysis indicated that the differentially expressed lipid metabolites were mainly associated with glycerophospholipid metabolism, glycerolipid metabolism, arachidonic acid metabolism, cholesterol metabolism, and fatty acid degradation. The classification of differentially expressed lipids was based on pathway types in the KEGG database. Figure 6a shows the enrichment pathways of differentially expressed lipids that accounted for more than 10% of all differentially expressed lipids. Among these, glycerophospholipid metabolism had 275 significantly differentially expressed lipid metabolites, accounting for 65.01% of all differentially expressed lipids; retrograde endocannabinoid signaling had 152 lipid metabolites, accounting for 35.93%; choline metabolism in cancer had 135 lipid metabolites, accounting for 31.91%; glycerolipid metabolism had 124 lipid metabolites, accounting for 29.31%; fat digestion and absorption had 114 lipid metabolites, accounting for 26.95%. The enrichment analysis of differentially expressed lipids is shown in Figure 6b, revealing significant differences in the glycerophospholipid metabolism, choline metabolism in cancer, arachidonic acid metabolism, and retrograde endocannabinoid signaling pathways (*p* < 0.05).

## 4. Discussion

### 4.1. Method Development

This study developed a quantitative analysis method for HFPO-DA in various mouse tissues. Previous detection methods mainly focused on measuring HFPO-DA in environmental samples such as water, soil, and plants, with a few serum sample analyses [6,30]. An EC-C18 chromatography column with a 2.7 μm particle size effectively separated the target compound within five minutes, significantly improving the efficiency of tissue sample analysis. The incorporation of minute quantities of formic acid into the mobile phase enhanced the chromatographic peak shape and instrument responsiveness and decreased the sample analysis cycle [31]. Hydroxymethyl coumarin was selected as the IS due to its similarity to the target compound in instrument response and elution time, as well as its excellent stability [32]. When preparing the reference solution, mixing hydroxymethyl coumarin with HFPO-DA did not interfere with the detection of HFPO-DA. UPLC-MS/MS enabled the accurate quantification of multiple compounds. In the sample analysis, HFPO-DA was separated effectively within seven minutes. Proteins were precipitated directly during sample processing using three times the volume of methanol. This method minimized matrix effects, ensured high extraction recovery rates, and provided good reproducibility and operational simplicity. Methodological validation studies showed that the detection method performed well in terms of selectivity, linearity, precision, accuracy, mechanism effects, and recovery [33]. Sample stability testing under three storage conditions and various dilution factors indicated good stability.

### 4.2. Pharmacokinetics and Tissue Distribution

HFPO-DA is a short-chain perfluoroalkyl substance akin to perfluorocarboxylic acids. Its strong carbon-fluorine bonds resist metabolism in the body [34]. This experiment measured HFPO-DA levels in mouse serum and tissues at various time points after a single 30 mg·kg^−1^ oral dose and after repeated 3 mg·kg^−1^ oral doses over 16 days. Following single oral exposure, HFPO-DA levels in serum and seven types of tissue were measured at 10 time points. Fat tissue was divided into WAT and BAT based on their different functional roles [35]. NCA showed that after oral administration, the serum concentration-time curve of HFPO-DA peaked once at 0.5 h, indicating rapid absorption and tissue entry; this was consistent with previous reports [15]. Its elimination half-life in serum was 7.95 h, and its clearance rate was 330.06 mL·h^−1^·kg^−1^, significantly higher than that of PFOA and PFOS [36]. The pharmacokinetics of HFPO-DA were found to vary according to sex: it was absorbed more slowly in male serum (T_max_ 1.0 h) compared to female serum (T_max_ 0.5 h), with higher peak concentrations in males (C_max_ 9.23 μg·mL^−1^) than in females (C_max_ 9.00 μg·mL^−1^). HFPO-DA was eliminated more slowly from males (t_1/2_ 8.23 h) than from females (t_1/2_ 7.54 h). This suggested that HFPO-DA has a greater and longer-lasting effect on males than females. This may be due to PFASs having effects related to sex hormones [37,38,39].

Studying the spread of toxic substances in various tissues and organs throughout the body is vital for identifying target organs and potential accumulation sites [40]. This study revealed the distribution of HFPO-DA in critical mouse tissues over time. The results indicated that HFPO-DA was present in all eight tested tissue types, aligning with the high distribution volume observed in the serum (V_z/F_ 3783.25 mL·kg^−1^). This suggested the substance has a strong affinity for tissues and a potential for accumulation. Further statistical analysis showed that HFPO-DA rapidly entered tissues via the bloodstream, with the concentration peaking within 0.5 to 1 h. The concentration patterns over time were consistent across various tissues. The peak activity times for brain, white fat, and brown fat tissues were slightly delayed, possibly due to poor blood supply. Although the brain is not the primary organ where PFAS accumulates, compared to the blood and liver, studies have shown that PFAS can impair brain function [41]. This may be related to the disruption of tight junctions associated with the blood–brain barrier (BBB) [42]. The present study indicated that, despite its low levels in brain tissue, HFPO-DA can cross the BBB, potentially explaining its direct impact on the brain. Comparing different tissues, the liver and lungs had the highest content of HDPO-DA, consistent with previous reports indicating higher liver content [36].

After the continuous exposure of mice to HFPO-DA for 16 days, the liver maintained the highest content of HFPO-DA. This suggests that the liver may be the target organ affected by HFPO-DA, based on the dose. Notably, the lungs also had a very high HFPO-DA content, with a median concentration that surpassed that of the liver. This cumulative effect indicates that potential toxic risks to the lungs should not be overlooked. Previous studies have shown that many drugs that accumulate in the lungs, such as amiodarone and bleomycin, typically initiate a pathological process characterized by inflammatory infiltration and oxidative stress. This process may ultimately progress to a series of toxic reactions, including fibrosis or respiratory dysfunction [43,44,45]. Studies indicate that PFAS exposure can activate inflammasomes in bronchial epithelial cells, leading to the release of pro-inflammatory cytokines (IL-1β, IL-18, and IL-33) and airway hyperresponsiveness [46]. PFAS also promotes the proliferation of macrophages and lung epithelial cells, and is closely associated with the development and metastasis of lung cancer [47,48,49]. Based on the mechanisms of action observed in similar substances, it was hypothesized that HFPO-DA may cause lung tissue damage through pathways such as activating pulmonary macrophages, inducing oxidative stress, or triggering neutrophil infiltration. Detailed experiments and analyses on the effects of HFPO-DA on the lungs will be the subject of subsequent studies.

In repeated exposure experiments, although HFPO-DA was not detected in brain tissue, this did not necessarily mean that it did not enter the brain or had no effect on it. This finding may have been related to the adaptive response induced by repeated low-dose exposure, such as the induction of metabolic enzymes or the upregulation of efflux transporters [50,51]. Although HFPO-DA itself was rapidly cleared, its minute effects accumulated with each exposure, ultimately leading to functional outcomes. Additionally, while the detection method met conventional sensitivity standards, it may be insufficient to capture extremely low yet biologically significant concentrations resulting from repeated low-dose exposure. Therefore, the concentration of HFPO-DA within the organization cannot be relied upon as the sole indicator of neurotoxicity; the cumulative effect of functional changes may be more critical.

The gender-based analysis revealed that the distribution of HFPO-DA in tissues was similar in male and female mice. The time-concentration curve showed similarities in peak velocity, peak time, and elimination pattern. The concentration of HFPO-DA in male reproductive tissues was higher than in females, aligning with serum study data. This phenomenon reflects males’ reduced capacity to clear HFPO-DA, which may be related to the effects of androgens on the activity of certain metabolic enzymes in the liver, such as cytochrome P450 monooxygenase isozyme 3A4 (CYP3A4). Studies indicate that androgens can inhibit CYP3A4 activity, thereby decreasing the body’s ability to clear chemicals [52]. Additionally, studies indicate that PFAS are a class of endocrine-disrupting chemicals that can alter the expression of gender-related genes, increasing androgen levels while decreasing estrogen levels. This may further exacerbate the phenomenon of slower metabolism in males [53,54]. In a study examining serum levels of total PFAS exposure in humans, a similarly observed phenomenon was noted: clearance rates were lower in males than in females [55]. Therefore, the specific relationship and mechanisms underlying the metabolic differences and toxic effects of HFPO-DA in different mouse models warrant further investigation.

### 4.3. Lipidomics and KEGG Pathway Analysis

Lipidomics, a technique for analyzing the types and quantities of lipids, is widely utilized in research on metabolic diseases. It is also commonly used in environmental pollutant research but has rarely been used to study the effects of HFPO-DA on the liver [56]. This study examined changes in the liver-to-body weight ratio in mice following 16 consecutive days of receiving 3 mg·kg^−1^ HFPO-DA daily. The results suggested that exposure to HFPO-DA substantially increased the liver-to-body weight ratio in mice, indicating alterations in liver carbohydrate and lipid metabolism [57]. Targeted lipid metabolomics were then used to analyze and identify 1875 lipid metabolites across 43 lipids. Through the OPLS-DA model, 615 differentially expressed lipids were identified. The results confirmed that exposure to HFPO-DA is closely related to liver lipid metabolism disorders, consistent with previous reports. Previous studies have focused on the degree of unsaturation of lipids, and the present study also found that the impact of lipid chain length was very significant [58]. LCACs serve as biomarkers for mitochondrial function. In mitochondrial fatty acid degradation, LCFAs combine with carnitine to form LCACs through the action of carnitine palmitoyl transferase 1 (CPT1) and carnitine palmitoyl transferase 2 (CPT2). CPT2 is then transported into mitochondria for fatty acid beta-oxidation [59,60]. Following HFPO-DA treatment, LCACs and LCFAs significantly increased in the liver, indicating long-chain fatty acid metabolic disorders. This suggested that HFPO-DA may impact mitochondrial function in hepatocytes.

KEGG enrichment analysis showed that the differentially expressed lipids were significantly enriched in the glycerophospholipid and arachidonic acid metabolism pathways. In the arachidonic acid metabolic pathway, arachidonic acid is oxidized by cyclooxygenase (COX1/2) into lipid molecules that play roles in various physiological and pathological processes [61]. HFPO-DA treatment caused the accumulation of arachidonic acid, while its downstream products PGD2 and PGE2 were significantly reduced, which may have been related to its effects on COX1/2. Exposure to HFPO-DA also resulted in a significant increase in lysophosphatidic acid (LPA). This increase could accelerate the progression of diseases associated with macrophage dysfunction and inflammation. In the liver, high LPA levels can trigger severe inflammatory responses in Kupffer cells [62]. LPA can be phosphorylated by monoglyceride (MAG) through the monoglyceride kinase (MAGK) pathway, and MAG can originate from the degradation of triglycerides, which was consistent with the significant reduction in triglycerides observed [63]. This differed from prior lipid studies at drinking water concentrations, which may be due to the different HFPO-DA concentrations [64]. The decrease in triglycerides may also be related to the action of lipoprotein lipase (LPL) in the liver, which breaks triglycerides down into free fatty acids [65]. Following HFPO-DA exposure, both free cholesterol and cholesterol esters exhibited varying degrees of decreased expression. Following exposure to HFPO-DA, a noticeable reduction in free cholesterol and cholesterol esters was observed, potentially linked to the efflux and conversion of free cholesterol. ATP-binding cassette transporter A1 (ABCA1) is the main player in cholesterol efflux [66,67]. The conversion of cholesterol to bile acids is mainly regulated by cholesterol 7-alpha hydroxylase (CYP7A1) [68]. Activation of CYP7A1 indirectly inhibits acyl-CoA cholesterol acyltransferase (ACAT) [69,70], preventing cholesterol from being converted into cholesteryl esters.

**Figure 7:**
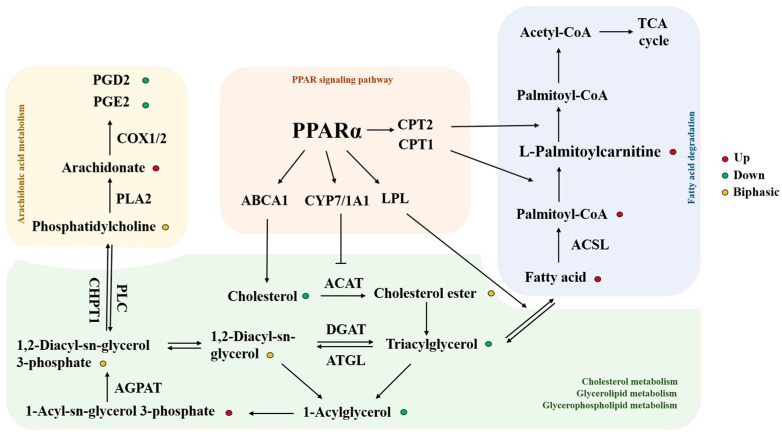
Molecular mechanisms underpinning lipid metabolism changes mediated by HFPO-DA. Red dots indicate significantly upregulated lipids; green dots signify significantly downregulated lipids; yellow dots indicate lipids that exhibited both upregulation and downregulation. Abbreviations: PPARα, peroxisome proliferator-activated receptor alpha; CPT1/2, carnitine palmitoyl transferase 1/2; ABCA1, ATP-binding cassette transporter A1; CYP7/1A1, cytochrome P450 7/1A1; LPL, lipoprotein lipase; ACAT, cholesterol acyltransferase; DGAT, diacylglycerol acyltransferase; ATGL, adipose triglyceride lipase; AGPAT, acylglycerolphosphate acyltransferase; PLC, phospholipase C; CHPT1, choline phosphotransferase 1; PLA2, phospholipase A2; COX1/2, mitochondrial cytochrome oxidase 1/2; ACSL, acyl-CoA synthetase long-chain family; TCA cycle, tricarboxylic acid cycle; PGE2, prostaglandin E2; PGD2, prostaglandin D2. →, promotion; –, inhibition.

Thus, these results revealed that HFPO-DA disrupts liver lipid metabolism by affecting multiple lipid-processing targets. Interestingly, most of the enzymes involved in differential lipid metabolism were closely related to the PPARα pathway. Therefore, from the perspective of lipid metabolism, PPARα could be the target for the effects of HFPO-DA [71]. Previous studies have demonstrated increased PPARα-dependent transcriptional signaling in mouse and human hepatocytes exposed to HFPO-DA [18,19,20]. In the HFPO-DA toxicity study, liver injury effects consistent with PPARα pathway activation were also observed, such as increased liver weight, hepatomegaly, and enhanced cellular proliferation [21,22,23]. These findings further corroborate the above hypothesis regarding the role of the PPARα pathway in the effects of HFPO-DA. Figure 7 illustrates the specific mechanism.

Furthermore, the lipidomics analysis revealed broader disturbances associated with other metabolic pathways. For example, the widespread downregulation of cholesterol esters, sphingomyelin, and ceramides strongly suggests a potential outcome resulting from the activation of the hepatic X receptor (LXR) pathway by HFPO-DA [72]. LXR is recognized as the master regulator of systemic cholesterol homeostasis. Its activation upregulates the expression of genes such as ABDCA1 and apolipoprotein E (ApoE), promoting cholesterol efflux and clearance [73]. Concurrently, LXR induces CYP7A1 expression, which converts cholesterol into bile acids, a process corroborated by the detection of significantly elevated chenodeoxycholic acid levels [74,75]. The simultaneous elevation of deoxycholic acid and significant upregulation of hemolytic phosphatidylcholine may be closely associated with the regulation of the farnesoid X receptor (FXR) pathway. The FXR pathway plays a central role in bile acid and lipid homeostasis [76]. These findings indicate that HFPO-DA may simultaneously interfere with multiple nuclear receptor signaling pathways, which coordinate with the PPARα pathway to form a complex lipid regulatory network.

It should be noted that the single (30 mg·kg^−1^) and repeat (3 mg·kg^−1^ for 16 days) dosages employed in this experiment were determined based on preliminary studies and previously reported acute toxicity tests. The primary objective was to identify the potential hazards and target organs of the environmental pollutant HFPO-DA, thereby providing direction for subsequent, more refined studies involving lower doses and prolonged exposure. However, it was found that HFPO-DA exposure levels as low as 3 mg·kg^−1^ can already simulate the levels of environmental exposure that exist in reality; as environmental pollution from HFPO-DA intensifies, this is a sobering finding [2].

The current study had some limitations. Due to blood volume constraints, continuous sampling from the same mouse was not feasible, necessitating the use of different mice at each time point. Sparse variable statistical analysis was employed, which did not provide a standard deviation, although it met the requirements for dynamic statistical analysis. This study examined the initial absorption and tissue distribution of HFPO-DA, laying the groundwork for research on its hepatotoxicity. It explored potential mechanisms of HFPO-DA hepatotoxicity by analyzing differences in lipid metabolites but did not assess dose dependence. Future studies will involve multi-dose interventions and further mechanism experiments to validate these hypotheses.

## 5. Conclusions

This study demonstrates that when mice are orally exposed to HFPO-DA, the compound is rapidly absorbed into the bloodstream and undergoes rapid and extensive distribution throughout tissues, exhibiting high accumulation concentrations in the liver and lungs. HFPO-DA induces liver lipid metabolism disorders through multi-target mechanisms, while the lungs—as a high-exposure target—present urgent health risks requiring assessment. HFPO-DA can cross the blood–brain barrier, suggesting potential neurotoxicity. These findings provide clear target-oriented guidance for the health risk assessment of HFPO-DA. Subsequent studies should focus on sensitive effect endpoints for hepatotoxicity, pulmonotoxicity, and neurotoxicity to establish a comprehensive evaluation framework. Testing for hepatotoxicity, pulmonotoxicity, and neurotoxicity should be mandated as requirements for HFPO-DA safety assessments, thereby providing more robust scientific safeguards for public health.

## Figures and Tables

**Table 1:** Standard curve and detection limits for HFPO-DA (*n* = 6).

Analyte	Linear Range (ng·mL^−1^)	Linear Regression Equation	Correlation Coefficient (r)	LLOQ (ng·mL^−1^)	RSD of LLOQ (%)	RE of LLOQ (%)
serum	2–1000	y = 1.52 × 10^4^x + 1.62 × 10^4^	0.9976	2	12.65	0.01

**Table 2:** Intra-day and inter-day precision and accuracy of HFPO-DA measurements (*n* = 6).

HFPO-DA	Concentration (ng·mL^−1^)	Intra-Day			Inter-Day		
		Mean ± SD (ng·mL^−1^)	RSD (%)	RE (%)	Mean ± SD (ng·mL^−1^)	RSD (%)	RE (%)
Low concentration	6	6.85 ± 0.34	4.97	14.10	6.84 ± 0.18	2.67	−2.27
Medium concentration	400	394.27 ± 5.76	1.46	−1.43	395.43 ± 7.62	1.93	−1.14
High concentration	800	802.36 ± 9.49	1.18	0.30	800.07 ± 6.92	0.86	0.01

**Table 3:** Extraction recovery and matrix effect for HFPO-DA (*n* = 6).

HFPO-DA	Concentration (ng·mL^−1^)	Extraction Recovery		Matrix Effect	
		Mean (%)	RSD (%)	Mean (%)	RSD (%)
Low concentration	6	96.92 ± 3.21	3.31	96.97 ± 2.18	2.24
medium concentration	400	95.00 ± 1.28	1.35	96.81 ± 1.98	2.05
High concentration	800	97.76 ± 2.35	2.40	98.03 ± 1.27	1.29

**Table 4:** Stability of HFPO-DA under various conditions (*n* = 6).

Condition	Concentration (ng·mL^−1^)	Mean ± SD (ng·mL^−1^)	RSD (%)	RE (%)
Storage at room temperature for five hours	6	6.82 ± 0.26	3.81	13.71
	400	393.25 ± 5.21	1.33	−1.69
	800	797.22 ± 13.10	1.64	−0.35
Storage at −80 °C for 15 days	6	6.88 ± 0.29	4.20	14.71
	400	396.80 ± 6.87	1.73	−0.80
	800	806.89 ± 10.03	1.24	0.86
Three freeze–thaw cycles	6	6.73 ± 0.22	3.32	12.19
	400	389.53 ± 13.49	3.46	−2.62
	800	802.12 ± 11.35	1.41	0.26

**Table 5:** HFPO-DA dilution integrity (*n* = 6).

Dilution	Concentration After Dilution (ng·mL^−1^)	Mean ± SD (ng·mL^−1^)	RSD (%)	RE (%)
1/25	160	153.45 ± 5.02	0.75	0.24
1/10	400	394.22 ± 6.20	3.27	−4.09
1/5	800	801.94 ± 5.98	1.57	−1.45

**Table 6:** Pharmacokinetic parameters after oral exposure to HFPO-DA at 30 mg·kg^−1^ (total *n* = 6, female/male *n* = 3).

Mice	C_max_ (ng·mL^−1^)	T_max_ (h)	AUC_(0-t)_ (h·ng·mL^−1^)	AUC_(0-∞)_ (h·ng·mL^−1^)	t_1/2_ (h)	CL_z/F_ (mL·h^−1^·kg^−1^)	V_z/F_ (mL·kg^−1^)
Total	9066.67	0.5	80,566.05	90,893.73	7.95	330.06	3783.25
Female	9000.00	0.5	69,272.50	76,438.65	7.54	392.47	4265.69
Male	9226.67	1.0	91,859.61	105,429.62	8.23	284.55	3379.24

**Table 7:** Pharmacokinetic parameters for each tissue after oral exposure to 30 mg·kg^−1^ HFPO-DA (total *n* = 6, female/male *n* = 3).

Analyte	Mouse	C_max_ (ng·g^−1^)	T_max_ (h)	AUC_(0-t)_ (h·ng·g^−1^)	t_1/2_ (h)
Heart	Total	3815.96	0.50	20,350.10	3.67
	Female	3627.05	0.50	13,012.38	3.29
	Male	4004.87	0.50	25,686.48	4.26
Liver	Total	10,877.13	0.50	44,324.97	4.43
	Female	8113.31	1.00	28,178.67	3.37
	Male	13,879.58	0.50	60,471.27	4.12
Spleen	Total	3542.87	0.50	17,731.19	2.76
	Female	3243.90	0.50	18,624.10	1.75
	Male	4237.26	1.00	16,838.28	4.03
Lung	Total	6508.74	0.50	38,229.16	4.80
	Female	6598.41	1.00	34,744.64	3.72
	Male	7710.97	0.50	41,713.68	4.69
Kidney	Total	4379.75	0.50	21,633.41	4.47
	Female	4237.26	1.00	16,838.28	4.03
	Male	4917.66	0.50	26,428.54	4.41
Brain	Total	287.10	0.50	934.90	4.28
	Female	264.56	1.00	548.26	- *
	Male	321.85	0.50	1276.86	9.00
WAT	Total	2345.75	1.00	11,786.75	1.90
	Female	2587.39	1.00	9771.23	3.87
	Male	2104.11	1.00	12,120.13	2.36
BAT	Total	2952.58	0.50	16,224.88	3.90
	Female	2796.12	1.00	11,440.93	2.96
	Male	3412.95	0.50	21,008.84	4.34

* Due to low concentration, insufficient data were obtained, and no results could be calculated.

## Data Availability

The article and Supplementary Materials detail the study’s original contributions. The corresponding author is available for further inquiries.

## Supplementary Materials

The following supporting information can be downloaded at: https://www.mdpi.com/article/10.3390/toxics13100850/s1, Table S1: Standard curve and detection limits for HFPO-DA (*n* = 6); Table S2: Intra-day and inter-day precision and accuracy of HFPO-DA measurement (*n* = 6); Table S3: Extraction recovery and matrix effect for HFPO-DA (*n* = 6); Table S4: Stability of HFPO-DA under various conditions (*n* = 6); Table S5: HFPO-DA dilution integrity (*n* = 6); Table S6: Partially different lipid metabolites after the action of HFPO-DA.
