# Supplementary material for: Absorption and Tissue Distribution of Environmental Pollutant HFPO-DA, and Its Effect on Hepatic Lipid Metabolism Reprogramming in Mice
Source: Toxics. 2025 Oct 8;13(10):850. doi: 10.3390/toxics13100850 (PMC12568254; doi:10.3390/toxics13100850)
Supplement: Supplementary file 1 [file toxics-13-00850-s001.zip › Supplementary Materials (Table S1-Table S5)-edit.pdf]

Supplementary materials

Table S1. Standard curve and detection limits for HFPO-DA (n = 6).

**Analyte**	**Linear range ($ng \cdot mL^{-1}$)**	**Linear regression equation**	**Correlation coefficient (r)**	**LLOQ ($ng \cdot mL^{-1}$)**	**RSD of LLOQ (%)**	**RE of LLOQ (%)**
Heart	2-1,000	$y = 2.2 \times 10^4 x - 388$	0.9951	2	4.23	2.93
Liver	2-1,000	$y = 1.96 \times 10^4 x + 2.97 \times 10^5$	0.9947	2	8.38	1.63
Spleen	1-1,000	$y = 1.84 \times 10^4 x + 3.26 \times 10^5$	0.9970	1	13.36	2.92
Lungs	2-1,000	$y = 2.04 \times 10^4 x + 2.53 \times 10^5$	0.9946	2	13.36	4.40
Kidney	2-1,000	$y = 1.98 \times 10^4 x + 4.52 \times 10^5$	0.9984	2	13.07	3.67
Brain	3-1,000	$y = 2.06 \times 10^4 x + 2.24 \times 10^5$	0.9946	3	9.44	-3.33
fat	2-1,000	$y = 1.94 \times 10^4 x + 1.62 \times 10^6$	0.9931	2	10.25	0.79

Table S2. Intra-day and inter-day precision and accuracy of HFPO-DA measurement (n = 6).

Samples	HFPO-DA	Intra-day			Inter-day		
		Mean ± SD ($ng \cdot mL^{-1}$)	RSD (%)	RE (%)	Mean ± SD ($ng \cdot mL^{-1}$)	RSD (%)	RE (%)
**Heart**	Low concentration	6.81 ± 0.30	4.43	13.50	6.70 ± 0.08	1.24	7.03
	medium concentration	396.20 ± 7.60	1.92	-0.95	396.51 ± 6.07	1.53	-0.87
	High concentration	802.97 ± 7.06	0.88	0.37	800.35 ± 7.78	0.97	0.04
**Liver**	Low concentration	6.85 ± 1.02	14.82	14.24	6.72 ± 0.62	9.29	-5.31
	medium concentration	408.97 ± 24.10	5.89	2.24	425.15 ± 25.05	5.89	-3.21
	High concentration	807.44 ± 15.11	1.87	0.93	805.69 ± 10.52	1.31	-1.50
**Spleen**	Low concentration	6.98 ± 0.62	8.83	16.40	6.31 ± 0.36	5.68	5.23
	medium concentration	401.59 ± 9.68	2.41	0.40	399.07 ± 39.01	9.77	-0.23
	High concentration	802.87 ± 16.16	2.01	0.36	803.25 ± 15.06	1.88	0.41
**Lung**	Low concentration	7.07 ± 0.61	8.68	17.85	6.80 ± 0.16	2.41	13.34
	medium concentration	396.00 ± 12.33	3.11	-1.00	431.30 ± 22.70	5.26	7.83
	High concentration	803.52 ± 24.87	3.10	0.44	789.87 ± 23.29	2.95	-1.27
**Kidney**	Low concentration	6.84 ± 0.14	2.04	13.99	6.64 ± 0.43	6.48	10.64
	medium concentration	397.68 ± 7.93	1.99	-0.58	404.77 ± 15.02	3.71	1.19
	High concentration	829.52 ± 26.05	3.14	3.69	808.21 ± 19.66	2.43	1.03
**Brain**	Low concentration	6.60 ± 0.65	9.78	10.06	6.96 ± 0.16	2.29	16.00
	medium concentration	397.56 ± 4.80	1.21	-0.61	402.12 ± 7.39	1.84	0.53
	High concentration	795.08 ± 34.87	4.39	-0.61	801.16 ± 27.81	3.47	0.14
**Fat**	Low concentration	6.31 ± 0.71	11.30	5.19	6.49 ± 0.53	8.18	8.15
	medium concentration	395.89 ± 14.11	3.56	-1.03	401.72 ± 24.66	6.14	0.43
	High concentration	795.71 ± 13.86	1.74	-0.54	808.15 ± 19.73	2.44	1.02

Table S3. Extraction recovery and matrix effect for HFPO-DA (n = 6).

Samples	HFPO-DA	Extraction recovery		Matrix effect	
		Mean (%)	RSD (%)	Mean (%)	RSD (%)
**Heart**	Low concentration	99.22 ± 1.71	1.72	96.58 ± 2.19	2.27
	medium concentration	96.06 ± 2.58	2.69	98.49 ± 1.56	1.58
	High concentration	96.96 ± 2.99	3.09	96.61 ± 3.25	3.37
**Liver**	Low concentration	97.54 ± 3.36	3.44	94.59 ± 1.87	1.98
	medium concentration	96.51 ± 5.43	5.63	98.27 ± 5.51	5.61
	High concentration	95.00 ± 1.20	1.27	98.29 ± 3.91	3.98
**Spleen**	Low concentration	94.58 ± 6.00	6.34	97.51 ± 6.01	6.16
	medium concentration	98.63 ± 4.66	4.72	99.18 ± 3.67	3.70
	High concentration	100.04 ± 2.46	2.46	99.19 ± 2.30	2.32
**Lung**	Low concentration	101.12 ± 2.97	2.94	97.82 ± 6.08	6.22
	medium concentration	95.23 ± 3.10	3.26	100.58 ± 4.32	4.29
	High concentration	95.78 ± 2.56	2.67	97.16 ± 1.71	1.76
**Kidney**	Low concentration	95.79 ± 3.35	3.50	100.70 ± 7.40	7.35
	medium concentration	103.81 ± 5.00	4.81	94.99 ± 4.00	4.21
	High concentration	96.98 ± 3.67	3.78	96.73 ± 4.03	4.17
**Brain**	Low concentration	99.93 ± 2.01	2.02	96.22 ± 7.91	8.22
	medium concentration	95.65 ± 2.57	2.69	93.97 ± 4.93	5.25
	High concentration	96.23 ± 3.61	3.76	96.57 ± 3.76	3.89
**Fat**	Low concentration	95.07 ± 2.21	2.32	95.90 ± 2.47	2.58
	medium concentration	95.51 ± 3.56	3.73	96.00 ± 2.54	2.64
	High concentration	95.71 ± 3.65	3.82	96.01 ± 3.02	3.15

Table S4. Stability of HFPO-DA under various conditions (n = 6)

Samples	Condition	Concentration (ng·mL⁻¹)	Mean ±SD (ng·mL⁻¹)	RSD (%)	RE (%)
**Heart**	Storage at room temperature for five hours	6	6.83 ± 0.33	4.97	13.96
		400	395.10 ± 6.22	1.58	-1.22
		800	807.84 ± 7.99	0.99	0.98
	Storage at –80 °C for 15 days	6	6.89 ± 0.28	4.14	14.96
		400	399.52 ± 7.54	1.89	-0.12
		800	811.10 ± 7.33	0.90	1.39
	Three freeze-thaw cycles	6	6.92 ± 0.19	2.86	15.49
		400	385.03 ± 5.17	1.35	-3.74
		800	799.24 ± 2.49	0.31	-0.09
Liver	Storage at room temperature for five hours	6	6.93 ± 0.51	7.49	15.61
		400	392.28 ± 28.84	7.35	-1.93
		800	773.33 ± 15.73	2.03	-3.33
	Storage at –80 °C for 15 days	6	6.90 ± 0.50	7.25	15.16
		400	404.64 ± 21.58	5.33	1.16
		800	821.82 ± 41.91	5.10	2.73
	Three freeze-thaw cycles	10	6.64 ± 0.68	10.36	10.76
		400	377.37 ± 20.27	5.37	-5.66
		800	811.16 ± 26.92	3.32	1.40
Spleen	Storage at room temperature for five hours	6	6.67 ± 0.61	9.23	11.19
		400	390.46 ± 17.7	4.53	-2.38
		800	816.19 ± 30.52	3.74	2.02
	Storage at –80 °C for 15 days	6	6.51 ± 0.30	4.73	8.55
		400	379.88 ± 14.67	3.86	-5.03
		800	781.15 ± 37.76	4.83	-2.36
	Three freeze-thaw cycles	6	6.35 ± 0.45	7.18	5.92
		400	375.17 ± 24.01	6.40	-6.21
		800	801.59 ± 16.79	2.10	0.20
Lung	Storage at room temperature for five hours	6	6.85 ± 0.28	4.17	14.23
		400	377.14 ± 16.47	4.37	-5.71
		800	781.19 ± 22.05	2.82	-2.35
	Storage at –80 °C for 15 days	6	7.08 ± 0.48	6.84	18.05
		400	386.7 ± 15.64	4.05	-3.32
		800	788.73 ± 30.24	3.83	-1.41
	Three freeze-thaw cycles	6	6.65 ± 0.26	3.98	10.90
		400	374.43 ± 11.15	2.98	-6.39
		800	795.8 ± 33.61	4.22	-0.52
Kidney	Storage at room temperature for five hours	6	6.74 ± 0.33	4.93	12.44
		400	405.61 ± 15.4	3.80	1.40
		800	790.88 ± 28.4	3.59	-1.14
	Storage at –80 °C for 15 days	6	7.07 ± 0.37	5.35	17.94
		400	392.48 ± 11.5	2.93	-1.88
		800	831.76 ± 27.34	3.29	3.97
	Three freeze-thaw cycles	6	6.54 ± 0.25	3.87	9.15
		400	398.70 ± 5.14	1.29	-0.32
		800	810.49 ± 29.9	3.69	1.31

Brain	Storage at room temperature for five hours	6	6.86 ± 0.68	10.04	14.43
		400	393.38 ± 18.19	4.63	-1.65
		800	791.10 ± 36.85	4.66	-1.11
	Storage at –80 °C for 15 days	6	6.82 ± 0.61	9.08	13.72
		400	400.31 ± 22.32	5.58	0.08
		800	798.66 ± 39.94	5.00	-0.17
	Three freeze-thaw cycles	6	6.40 ± 0.31	5.00	6.72
		400	402.04 ± 4.68	1.16	0.51
		800	768.79 ± 38.61	5.02	-3.90
Fat	Storage at room temperature for five hours	6	6.16 ± 0.61	10.04	2.73
		400	378.23 ± 17.36	4.59	-5.44
		800	780.86 ± 36.38	4.66	-2.39
	Storage at –80 °C for 15 days	6	6.39 ± 0.48	7.55	6.59
		400	396.91 ± 12.45	3.14	-0.77
		800	819.30 ± 31.67	3.87	2.41
	Three freeze-thaw cycles	6	6.59 ± 0.38	5.84	9.85
		400	396.99 ± 6.55	1.65	-0.75
		800	810.98 ± 23.17	2.86	1.37

Table S5. HFPO-DA dilution integrity (n = 6).

Samples	Dilution	Concentration after dilution (ng·mL^{-1})	Mean ± SD (ng·mL^{-1})	RSD (%)	RE (%)
**Heart**	1/25	160	157.88 ± 4.42	1.18	0.24
	1/10	400	399.48 ± 8.91	2.80	-1.32
	1/5	800	801.91 ± 9.47	2.23	-0.13
**Liver**	1/25	160	161.74 ± 3.43	0.80	0.31
	1/10	400	396.50 ± 5.50	2.12	1.09
	1/5	800	802.46 ± 6.42	1.39	-0.87
**Spleen**	1/25	160	153.82 ± 4.84	5.98	0.01
	1/10	400	384.02 ± 8.06	3.15	-3.86
	1/5	800	800.11 ± 47.86	2.10	-3.99
**Lung**	1/25	160	155.11 ± 4.74	4.41	1.28
	1/10	400	385.00 ± 14.26	3.06	-3.06
	1/5	800	810.24 ± 35.74	3.70	-3.75
**Kidney**	1/25	160	158.25 ± 5.37	4.11	-0.10
	1/10	400	385.84 ± 21.07	3.39	-1.09
	1/5	800	799.17 ± 32.83	5.46	-3.54
**Brain**	1/25	160	159.15 ± 2.47	1.77	-0.44
	1/10	400	393.83 ± 6.74	1.56	-0.53
	1/5	800	796.45 ± 14.11	1.71	-1.54
**Fat**	1/25	160	149.60 ± 6.27	2.15	0.53
	1/10	400	400.64 ± 5.11	4.19	-6.50
	1/5	800	804.21 ± 17.32	1.27	0.16
